# Inhibition of NOS-NO System Prevents Autoimmune Orchitis Development in Rats: Relevance of NO Released by Testicular Macrophages in Germ Cell Apoptosis and Testosterone Secretion

**DOI:** 10.1371/journal.pone.0128709

**Published:** 2015-06-05

**Authors:** Sabrina Jarazo Dietrich, Mónica Irina Fass, Patricia Verónica Jacobo, Cristian Marcelo Alejandro Sobarzo, Livia Lustig, María Susana Theas

**Affiliations:** Instituto de Investigaciones Biomédicas (UBA-CONICET), Facultad de Medicina, Universidad de Buenos Aires, Buenos Aires, Argentina; Zhejiang University, CHINA

## Abstract

**Background:**

Although the testis is considered an immunoprivileged organ it can orchestrate immune responses against pathological insults such as infection and trauma. Experimental autoimmune orchitis (EAO) is a model of chronic inflammation whose main histopathological features it shares with human orchitis. In EAO an increased number of macrophages infiltrate the interstitium concomitantly with progressive germ cell degeneration and impaired steroidogenesis. Up-regulation of nitric oxide (NO)-NO synthase (NOS) system occurs, macrophages being the main producers of NO.

**Objective:**

The aim of our study was to evaluate the role of NO-NOS system in orchitis development and determine the involvement of NO released by testicular macrophages on germ cell apoptosis and testosterone secretion.

**Method and Results:**

EAO was induced in rats by immunization with testicular homogenate and adjuvants (E group) and a group of untreated normal rats (N) was also studied. Blockage of NOS by i.p. injection of E rats with a competitive inhibitor of NOS, L-NAME (8mg/kg), significantly reduced the incidence and severity of orchitis and lowered testicular nitrite content. L-NAME reduced germ cell apoptosis and restored intratesticular testosterone levels, without variations in serum LH. Co-culture of N testicular fragments with testicular macrophages obtained from EAO rats significantly increased germ cell apoptosis and testosterone secretion, whereas addition of L-NAME lowered both effects and reduced nitrite content. Incubation of testicular fragments from N rats with a NO donor DETA-NOnoate (DETA-NO) induced germ cell apoptosis through external and internal apoptotic pathways, an effect prevented by N-acetyl-L-cysteine (NAC). DETA-NO inhibited testosterone released from Leydig cells, whereas NAC (from 2.5 to 15 mM) did not prevent this effect.

**Conclusions:**

We demonstrated that NO-NOS system is involved in the impairment of testicular function in orchitis. NO secreted mainly by testicular macrophages could promote oxidative stress inducing ST damage and interfering in Leydig cell function.

## Introduction

Male genital tract inflammation, mainly orchitis and orchi-epididymitis, are relevant co-factors of human subfertility and infertility. In testicular biopsies of patients with orchitis, infiltration of lymphocytes and macrophages is frequently found associated with damage of seminiferous tubules (ST) resulting in focal or severe alterations of spermatogenesis. In most cases, inflammation also involves the epididymis resulting in orchi-epididymitis [1,2]. Importantly, infiltrating immune cells can produce a pro-inflammatory microenvironment that might be responsible for impairment of spermatogenesis in orchitis. Infiltrating immune cells not only synthesize pro-inflammatory cytokines Th1 (IL-6, TNF-α IFN-γ) and Th17 (IL-17, IL-21 and IL-23) but also produce pro-oxidative species formed from oxygen and/or nitrogen like hydrogen peroxide and nitric oxide (NO). Increased expression of NO synthase (NOS) has been described in the testis of infertile patients and oxidative stress proposed as a detrimental condition for male reproductive health [3–5].

Nitric oxide (NO) is a pro-oxidative molecule with multiple biological actions synthesized by enzymatic conversion of L-arginine to L-citrulline catalysed by NOS. In general, low concentrations of NO (<1μM) promote cell survival, proliferation and homeostasis, whereas higher levels (>1μM) such as occur during inflammatory processes generate oxidative stress favoring cell cycle arrest, apoptosis, and senescence [6]. Although NO was suggested as the main factor responsible for testicular oxidative stress, data on the effect and mechanism of action of NO on testicular function is lacking.

Experimental autoimmune orchitis (EAO) is a useful established model [7] to study mechanisms involved in pathological alteration of the testis associated with a chronic inflammatory process, which shares many features with human orchitis. We induced orchitis in rats by active immunization with testis homogenate and adjuvants [8]. Fifty days after the first immunization initial signs of testicular alterations were observed (focal EAO); testicular histopathology was characterized by interstitial lymphomononuclear cell infiltration (mainly macrophages, dendritic cells and T lymphocytes) and damage of ST which exhibited germ cell apoptosis and sloughing (mainly spermatocytes and spermatids), associated with alterations of blood-testis barrier (BTB) permeability and function [9–11]. Eighty days after the first immunization severe and extended seminiferous tubule damage and increased immune cell infiltration occurred with fibrosis, testicular atrophy and infertility. Also, Leydig cells showed hyperplasia and hypertrophy associated with increased intratesticular testosterone levels [12].

We previously described that in rats with EAO, the increased number of macrophages infiltrating the testis generate high levels of NO and pro-inflammatory cytokines (TNF-α, IL-6, Fas L and IFN-γ) which play a relevant role in germ cell survival and steroidogenesis [13,14]. High levels of NO generated by up-regulation of NO synthase (NOS) activity and expression are found concomitantly with the main alterations observed at the ST [14]. The aim of the present work was to ascertain the role of NO-NOS system in germ cell apoptosis and testosterone production by Leydig cells during development of a chronic inflammatory process associated with infertility; particularly highlighting the relevance of NO released by testicular macrophages in the paracrine control of apoptosis and steroidogenesis.

## Materials and Methods

### Animals

Male adult *Sprague-Dawley* rats 50–60 days old were purchased from Bioterio Central, Facultad de Farmacia y Bioquímica (Buenos Aires, Argentina). Animals were kept on a 22°C and 14h light, 10h dark schedule and fed standard food pellets and water ad libitum. Animal handling and experimentation followed the NIH Guide for the Care and Use of Experimental Animals and was approved by the local committee (CICUAL, Medical School, University of Buenos Aires).

### In vivo experiments

**Fig 1:**
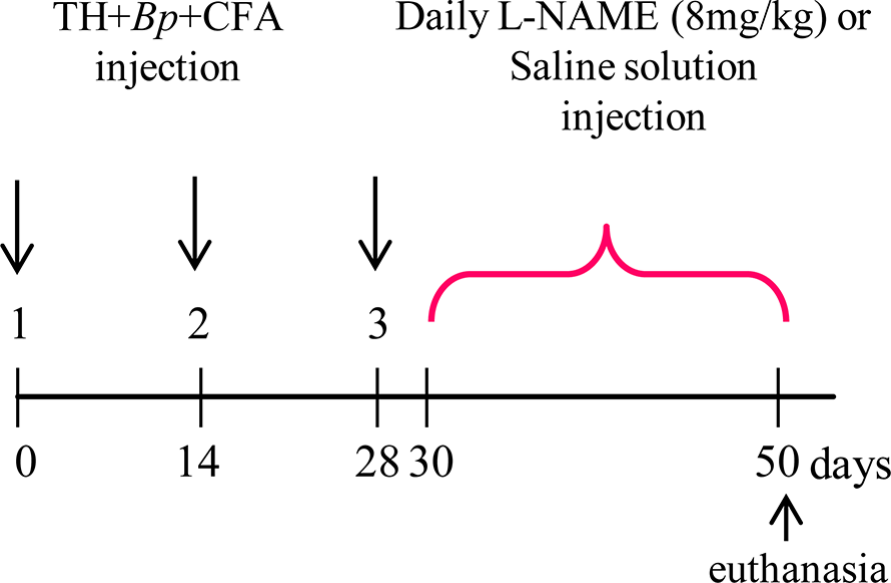
Induction of EAO and NOS blockage. Rats were actively immunized with testicular homogenate (TH), *Bordetella Pertussis* (*Bp*) and complete Freund´s Adjuvant (CFA) (for details see Material and Methods). 30 days after first immunization a group of rats received L-NAME (n = 12) and another saline solution (n = 11).

Induction of EAO and experimental designTo induce orchitis rats were actively immunized under anesthesia (100 mg/kg ketamine and 5 mg/kg xylazine) (n = 23) with testicular homogenate (TH) and adjuvants prepared as previously described (experimental group, E) (8). Briefly, rat testes were decapsulated, diluted in an equal volume of saline and disrupted in an Omni mixer for 30s. Final concentration was 500 mg/ml wet weight. A total of 0.4 ml of TH emulsified with 0.4 ml of complete Freund’s adjuvant (CFA) (Sigma-Aldrich, St. Louis, MO, USA) was injected intradermally in footpads and at multiple sites near ganglionar regions three times at 14 day intervals (day 0, day 14 and day 28) (Fig 1). The first two immunizations were followed by i.v. injection of 0.5 ml of *Bordetella pertussis* (Bp) (strain 10536; Instituto Malbrán, Buenos Aires, Argentina) containing 10^10^ inactivated microorganisms and the third by i.p. injection of microorganisms at a concentration of 5x10^9^ in saline solution. The incidence of orchitis was 67%. A group of normal, non immunized rats (N) was also studied All groups of rats were euthanized with CO_2_ 50days after the first immunization since at this time focal orchitis develops in E group. Body weight was determined and testes were removed and separated from epididymis and weighed. One testis was evaluated by histopathology and the other one was used to isolate macrophages. N rats were used to isolate testicular macrophages or to obtain testicular fragments.

To block NOS activity a group of immunized rats (E group) was i.p injected daily with the competitive NOS inhibitor, L-NAME (8 mg/kg, n = 12) and another group of E rats was injected daily with physiological solution (treatment control, n = 11) for 20 days from day 30 after the first immunization (previous to the appearance of testis lesions) (Fig 1). The following three groups each comprising 9–12 rats/group were studied: 1) E rats injected with saline (E+saline); 2) E rats injected with L-NAME (E+L-NAME); 3) normal, nonimmunised (N) rats. E rats were euthanized 50 days after the first immunization and N rats at the same time. One testis was fixed in Bouin’s solution and embedded in paraffin for histopathology evaluation and TUNEL technique and the other testis was kept at -70°C for nitrite and intratesticular testosterone determination. Blood was collected and sera were stored at -70°C for hormone assays.

#### Histopathology and EAO score

Transversal sections of equatorial area and poles of one testis from E+saline, E+L-NAME and N rats were analyzed. The degree of orchitis was scored as previously described [15]. Briefly, the score evaluates: a) the percentage of damaged seminiferous tubules (ST); (b) the degree of damage (sloughing and/or presence of degenerating germ cells); and (c) the testicular body weight ratio (T/Bw). EAO score = V+T+ P, where V is the value assigned to each percentage range of damaged ST (V = 0 (0–3%), 1 (3.1–4.9%), 2 (5–15.9%), 3 (16–25.9%), 4 (26–35.9%), 5 (36–55.9%), 6 (56–60.9%), 7 (61–79.9%), 8 (80–95.9%) and 9 (96–100%). T is an index of the degree of germ cell sloughing (T = 0: when mild germ cell sloughing and T = 0.5: only spermatogonia and Sertoli cells remain attached to ST walls). P is a correction factor for T/Bw (P = 0 or P = 0.5 when T/Bw was higher or lower than 2.5x10^-3^, respectively. EAO score above 2.5 indicates orchitis.

### In vitro experiments

#### Isolation of testicular macrophages

Cells from the testicular interstitium of N (n = 12) and E (n = 12) rats, were obtained as previously described (14). Briefly, decapsulated testes were incubated with Type I collagenase (0.3 mg/ml; Worthington Biochemical Corporation, Freehold, NJ, USA) in PBS containing 0.1% bovine serum albumin (BSA) (Sigma-Aldrich) at 34°C for 15 min in a Dubnoff shaking water bath. Collagenase was inactivated by adding ice-cold phosphate-buffered saline (PBS) and ST were allowed to settle for 2 min; the supernatant was washed with PBS and red blood cells were depleted by osmotic lysis with ammonium chloride (160 mM NH_4_Cl, 170 mM Tris-HCl, pH 7.2). Cells were washed, centrifuged at 300g for 10 min at 4°C and counted in a Neubauer chamber by the trypan blue exclusion method.

Macrophages were isolated from the interstitial cell suspension by Fluorescence-Activated Cell Sorting. Testicular interstitial cells (25x10^6^) were incubated with saturating concentrations of anti-OX42 (FITC) (125 μl direct, AbD Serotec, Oxford, UK) for 40 min at 26°C. Appropriate control isotypes were used. Stained cells were isolated by FACS in aseptic conditions (BD FACSAria II cytometer). Sorting gates were set on side scatter SSC/OX42 positive cell population. Cell debris was excluded by appropriate gate setting. Cells were sorted in Dulbecco´s Modified Eagle´s Medium-nutrient mixture F12 (DMEM/F12, Gibco-BRL, Rockville, MD, USA) containing 3% fetal bovine serum (FBS). After sorting, viability was tested by flow cytometry with propidium iodide (Sigma-Aldrich, 1mg/ml). Purity of sorted cells was 94.08%±1.02 (media±SEM) and viability 75.30%±2.41 (media±SEM), data from four independent experiments in which testes of 3 rats from each N and E group were used.

#### Isolation and culture of testicular fragments

Testicular fragments (TF) were obtained and cultured as previously reported [16]. Briefly, testes removed from N rats were decapsulated and carefully dissected with scissors into fragments about 3 mm^3^. TF were immediately fixed in Bouin’s solution to analyze the basal percentage of ST with apoptotic cells or placed onto a 0.4 μM pore size PET insert [(3 TF/insert/150μl), Becton, Dickinson and Company (BD), Frankling Lakes, NJ, USA] that allows diffusion of nutrients and molecules with a molecular weight lower than 20kDa. TF were cultured in DMEM/F12 medium supplemented (s) with L-glutamine (2 mM, Sigma-Aldrich), insulin-transferrin-selenium-A supplement (1x; ITS-A, Gibco BRL) and antibiotic-antimycotic solution (1x, Gibco BRL), and each insert was placed onto a 24-well culture plate (BD) in the presence or absence of testicular macrophages isolated by cell sorting for 18 h at 34°C in a humidified atmosphere with 5% CO_2_.

#### Co-cultures of testicular macrophages and testicular fragments

Testicular macrophages isolated by cell sorting from N and E rats were stabilized in DMEM/F12 medium containing 3% of FBS for 1h at 34°C in a humidified atmosphere with 5% CO_2_ and plated (1x10^6^) on a 24-well culture plate (BD) with DMEM/F12-s medium without phenol red in the presence or absence of a NOS inhibitor, L-nitro arginine methyl ester (L-NAME, 2 mM, Cayman Chemical, MI, USA) or of its inactive isomer D-NAME (2 mM, Cayman) for 1 h at 34°C in a humidified atmosphere with 5% CO_2_. Then the medium was replaced by the corresponding fresh medium and testicular macrophages were co-cultured with the TF obtained from N rats (n = 4) for 18 h (as described above). After the culture period, TF were fixed in Bouin’s solution and embedded in paraffin and apoptosis was evaluated in 4 μm thick non consecutive sections by the TdT-mediated dUTP nick end labeling (TUNEL) technique. Culture media were collected to evaluate NO content by a fluorometric kit (780051, Cayman Chemical Company) and testosterone by RIA.

#### Culture of testicular fragments in the presence of a nitric oxide donor

TF (3TF/well/300μl) from N rats (n = 5) were cultured onto 48-well culture plates (Corning Life Sciences-Axygen Inc, Union City, California, USA) with DMEM/F12-s medium with or without a NO donor DETA-NOate that releases NO at a slow rate over a prolonged period (*t*1/2 ~ 20 h) (DETA-NO, 1, 2 and 4 mM, Cayman Chemical Company) for 2, 4, 8 and 18 h. DETA-NO was dissolved 2 h before use in DMEM/F12-s medium to achieve a steady concentration of NO in solution. In the experiments performed to evaluate involvement of oxidative stress in NO induced germ cell apoptosis, TF were placed onto 48-well culture plates in DMEM/F12-s medium in the presence or absence of a general antioxidant, N-acetyl-L-cysteine (NAC, 1, 2.5, 5.0, 7.5 and 15.0 mM, Sigma-Aldrich) for 2 h at 34°C in a humidified atmosphere with 5% CO_2_. Then the medium was replaced by fresh medium containing or not DETA-NO (2 mM) or DETA-NO+NAC. After an 18h culture, TF were fixed in Bouin’s solution and embedded in paraffin and apoptosis evaluated by the TUNEL technique.

To evaluate the effect of NO and NAC on testosterone production TF (4TF/well/300μl) obtained from N rats (n = 5–8) were cultured as described above. After the culture period, the medium was collected, centrifuged and stored at -70°C until testosterone determination.

### Assessment of apoptosis

Apoptosis was determined by TUNEL assay. TF and testis sections (4 μm thick) were deparaffinized and hydrated by successive series of ethanol and then irradiated in a microwave oven (370W for 5 min) in 10 mM sodium citrate buffer, pH 6.0 and permeabilized with 0.1% Triton X-100 (Sigma-Aldrich) in 0.1% sodium citrate for 5 min at 4°C. Non-specific labeling was prevented by incubating sections with blocking solution (2% blocking reagent, Roche Molecular Biochemicals GmbH, Mannheim, Germany) in 150 mM NaCl and 100 mM maleic acid, pH 7.5 for 30 min at room temperature (RT). After 10 min incubation with terminal deoxynucleotidyl transferase (TdT) buffer (buffer TdT, 1x; CoCl_2_, 1x, Roche), apoptotic DNA was 3´-end labeled with digoxigenin-11-dideoxy-uridine triphosphate (4 μM Dig-ddUTP, Roche) by incubation with TdT (0.4 U/μl, Roche) in TdT buffer for 1 h at 37°C. As assay control, the TdT enzyme was replaced by the same volume of TdT buffer. Sections were then incubated with blocking solution (2% blocking reagent in 150 mM NaCl and 100 mM maleic acid, pH 7.5) for 30 min at RT, followed by detection of Dig-dd-UTP with an alkaline phosphatase-conjugated anti-digoxigenin antibody (0.375 mU/μl, Roche) incubated for 2 h at RT. Sections were rinsed and equilibrated in alkaline phosphatase buffer (100 mM Tris–HCl, 100 mM NaCl, 50 mM MgSO4, pH 9.5) containing 1 mM levamisole (Sigma-Aldrich). Then, alkaline phosphatase substrates, nitroblue tetrazolium and 5-bromo-4-chloro-3-indolyl-phosphate (NBT/BCIP, Roche) were added for 1 h. This reaction was stopped by washing with Tris–EDTA buffer (10 mM Tris–HCl, 1 mM EDTA, pH 8.0). Sections were rinsed in 95% ethanol for 24 h at RT, light counterstained with eosin, dehydrated by successive series of ethanol and mounted.

### Determination of lipid peroxides

To evaluate oxidative stress in the testis, we measured lipid hydroperoxidation by oxidation of Fe with the of xylenol Orange assay version 2 (FOX-2) [17]. This technique determines lipid hydroperoxidation without interference of non lipid hydroperoxides. Briefly, the assay is based on oxidation of ferrous (II) to ferric (III) ions by hydroperoxides present in a sample (in acid conditions). Ferric ions are complexed by the ferric ion indicator, xylenol orange (FOX), generating a blue-purple complex with an absorbance maximum of 550–600 nm. By addition of triphenylphosphine (TPP) lipid hydroperoxides are reduced, and cannot oxidize ferrous ions, and consequently the oxidative capacity of non lipid hydroperoxides is thereby determined. Briefly, testes (200 mg) of rats of N (n = 8) and E groups (n = 8) were homogenized in PBS using a potter's glass (200μl/g tissue). An aliquot was used to determine proteins by the Lowry method and lipids were extracted in cold methanol (5 ml/g tissue) with stirring conditions (1 min). Extracts were sonicated in ice (3 times 15 sec/each) and centrifuged at 10000g for 15 min at 4°C. Supernatants were used to evaluate lipid peroxidation. To determine hydroperoxide content, 50μl of the sample were incubated or not with 5.5μl of TPP 10mM in methanol for 30 min at RT and then incubated with 500ul of FOX solution (xylenol Orange 1mM, sulfate ferrous ammonium 2.5 mM, 2.6-di-tert-butyl-4-methylphenol 4.4mM, Sigma-Aldrich) for 30 min at RT in the dark. Finally 300μl of the sample (±TPP) were transferred to a 96 well plate and absorbance was measured at 570 nm in a microplate reader (model 550, Bio-Rad). Absorbance measured in the presence of TPP was subtracted from that obtained in absence of TPP. Lipid hydroperoxide concentrations were determined using a H_2_O_2_ standard calibration curve ranging from 3.125 to 50 μM.

### Western blotting

Caspases 3, 8, 9, and cytochrome c content were evaluated in cytosolic fraction and Bax and Bcl-2 in mitochondrial fraction by Western blot technique [18]. Briefly, TF obtained from N rats (n = 10) incubated with medium containing or not DETA-NO were homogenized with a glass homogenizer in buffer A (4 ml buffer/g tissue, 250 mM sucrose, 50 mM HEPES, 10 mM NaCl, 10 mM EDTA, 2 mM DTT, pH 7.4) with protease inhibitors (1 mM PMSF, 10 μg/ml leupeptin, 10 μg/ml pepstatin A and 10 μg/ml aprotinin, Sigma-Aldrich). Crude homogenates were centrifuged at 1000 × *g* for 10 min at 4°C and the supernatant centrifuged at 10,000 × *g* for 15 min at 4°C to sediment the low-speed fraction mainly containing mitochondria. Then the pellet with the mitochondrial fraction was diluted in mitochondrial lysis buffer (300 μl buffer/250 mg tissue, 20 mM HEPES, 1.5 mM MgCl_2_, 10 mM KCl, 1 mM EDTA, 1 mM EGTA and 1 mM DTT, pH 7.4) containing protease inhibitors. Tthe supernatant fraction resulting from 10,000 × *g* centrifugation was centrifuged at 100,000 × *g* for 60 min at 4°C, thereby obtaining the cytosolic fraction within the supernatant. To assess equal loading, protein concentration in lysates was determined by the Lowry method (Bio-Rad DC Protein Assay; Bio-Rad, Hercules, CA, USA). Before electrophoresis, samples were heated for 5 min at 95°C, then 25 μg of protein were resolved in SDS-polyacrylamide gel electrophoresis (PAGE) in a 15% denaturing gel at 120 V for 90 min at RT, then transferred to a PVDF membrane (Millipore, Bedford, MA, USA) for 1 h at 150 V at 4°C. Molecular weight of immunoreactive bands was determined by comparison to a ladder of pre-stained protein standards with a molecular weight range of 250–10 kDa (Precision Plus Protein Standards All Blue, Bio-Rad) applied to a line in each gel. Protein transference and equal loading were monitored by staining membranes with Ponceau red. Then, membranes were blocked with blocking solution (5% w/v) of non-fat dry milk in Tris buffered saline tween-20 buffer (TBST, 10 mM Tris, 154 mM NaCl, 0.1% Tween-20 (v/v), pH 7.5) for 90 min. Blots were probed overnight at 4°C with rabbit polyclonal antibody against caspase 9 (0.2 μg/ml, Santa Cruz Biotechnology, CA, USA), against caspase 3 (0.67 μg/ml R&D Systems, MN, USA) or caspase 8 (0.1 μg/ml, Santa Cruz), goat polyclonal antibody against cytochrome c (0.3 μg/ml, Santa Cruz), or mouse monoclonal antibodies against Bax or Bcl-2 (0.2 μg/ml, Santa Cruz). As internal loading control of the cytosolic fraction, a rabbit polyclonal antibody anti-actin (0.35μg/ml, Sigma-Aldrich) was used and a mouse monoclonal antibody anti-Oxphos Complex III core II subunit (COX III, 0.1μg/ml, Invitrogen, Corporation, Carlsbad, CA, USA) was used for mitochondrial fraction. After six washes in TBST (5 min each), membranes were incubated with an anti-mouse HRP (0.20 μg/ml, Chemicon International, Temecula, CA, USA) to detect COX III or anti-rabbit or anti-goat or anti- mouse biotinylated antibody (2.5 μg/ml, Vector Labs Burlingame, CA, USA) for the rest of the primary antibodies. Except for COX III, the reaction was enhanced with horseradish-streptavidin-peroxidase conjugates (0.33 μg/ml, Chemicon). Chemiluminescence was used to detect the horseradish-peroxidase-labelled protein. Bands were visualized in a G:Box Syngene system for imaging fluorescence and densitometrically quantified using Gene Tools software.

### Caspase 9 assay

Caspase 9 activity was measured in the cytosolic fraction of TF, obtained from N rats (n = 10) incubated with medium containing or not DETA-NO, following instructions provided by the Caspase-9 Colorimetric Assay Kit (APT 173, R&D). This assay is based on spectrophotometric detection of the chromophoro *p*-nitroniline (*p*-NA) after cleavage from the labeled substrate LEHD-*p*NA. Free *p*NA was quantified by a microplate reader at 405nm.

### Hormone determinations

LH, FSH and testosterone were measured by radioimmunoassay (RIA). Testosterone was evaluated in the culture medium, serum and tissue with Testo RIA-CT (DIA Source, Bélgica) and LH and FSH with a kit provided by the "National Hormone and Peptide Program” (Torrance, CA, USA). Testicular homogenates for determination of testosterone content were prepared as previously described [12]. The minimum detectable concentration of hormone in the assays was, testosterone: 0.1ng/ml, LH: 0.02ng/ml, FSH: 0.12ng/ml. Within-assay coefficient of variation was *<*8%.

### Nitric oxide determination

Nitrite, an indirect product of NO oxidation, was measured by Griess reaction [19]. Half a testis from E+saline (n = 11), E+L-NAME (n = 12) and N rats (n = 9) was decapsulated, homogenized and sonicated in Tris-HCl buffer containing 0.05% Triton X-100 (100 μl/g of tissue). The homogenate was centrifuged at 12,000×g for 30 min at 4°C and supernatant obtained; samples were deproteinized to prevent precipitates and turbidity by adding ZnSO_4_ (0.15 M, 10 μl/ml) and NaOH (10 M, 0.6 ml/ml) in Tris–HCl buffer 0.05% Triton X-100. The solution was stirred, incubated in ice for 15 min, then centrifuged at 12,000×g for 5 min at 4°C; the supernatant was used to evaluate nitrite content. To determine nitrite content, 100 μl of homogenate was applied to a 96-well culture plate, followed by 100 μl of Griess reagents (1% sulphanilamide in 5% phosphoric acid and 0.1% N-1-naphthylethylenediamine in equal parts, Sigma-Aldrich). After 15 min of color development at RT, absorbance was measured in a microplate reader at 595 nm (Model 550, Bio-Rad). Nitrite concentration was determined with a sodium nitrite standards calibration curve ranging from 1.57 to 25 μM.

### Statistical analysis

Data were expressed as mean±SEM. Results were analyzed by one or two way ANOVA followed by Bonferroni Multiple Comparison or Dunn Multiple Comparison Test. Western blot data were evaluated by One sample t Test. Data on nitrite content in conditioned media were evaluated by Student´s t Test. Statistical evaluations were considered significant if p < 0.05.

## Results

### NOS inhibition reduces the incidence and severity of EAO and preserves testicular function

At the end of treatment with NOS inhibitor the general condition of animals was good and there were no significant differences in body weight (E+saline: 393.3±13.09g, n = 11; E+L-NAME:383.4± 5.945g, n = 12; N: 390.10± 15.540g, n = 9).

**Fig 2:**
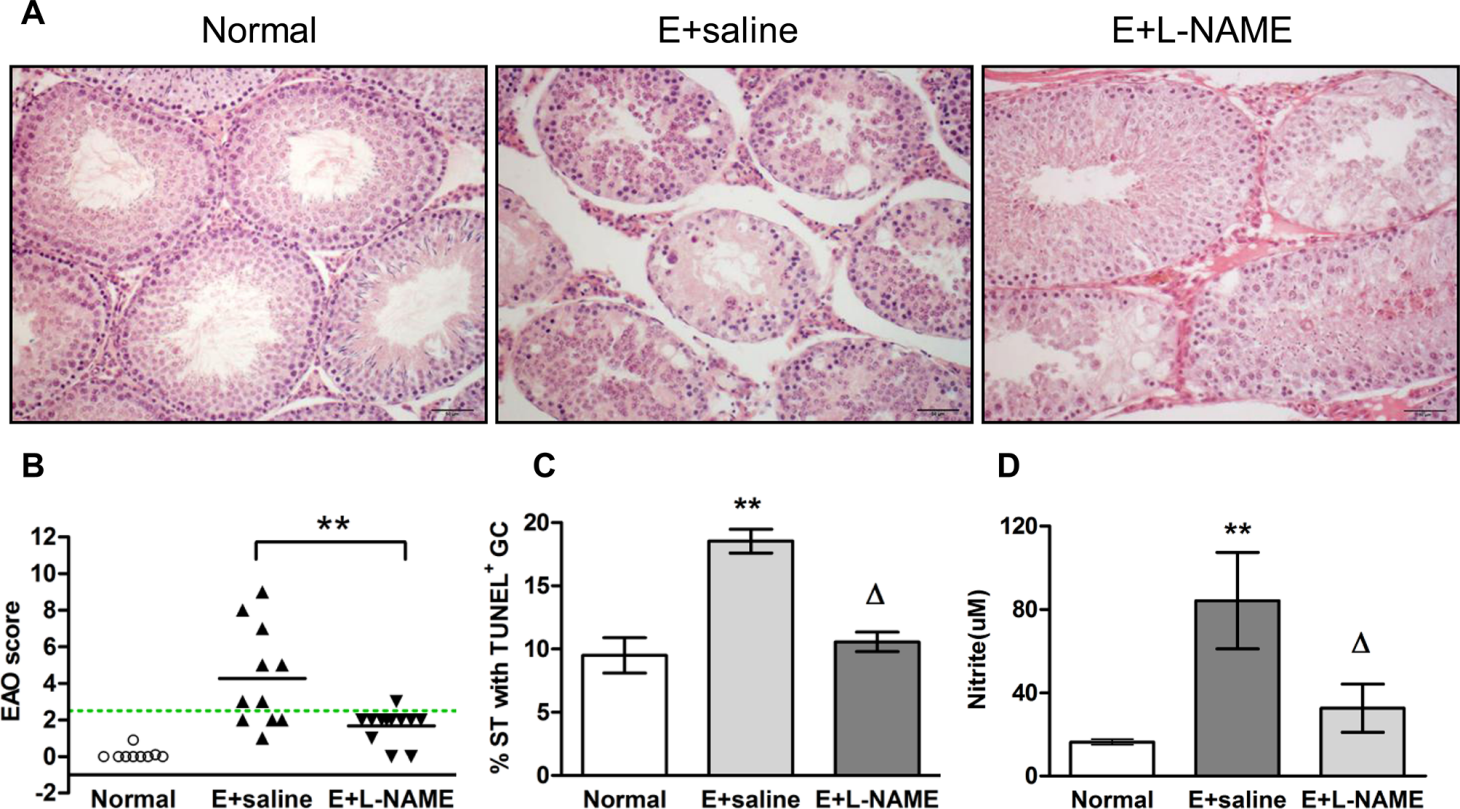
A: Testicular histopathology from normal (N, nonimmunized) and Experimental (E) rats (immunized with TH and adjuvants), injected with saline solution (E+saline) or L-NAME (8mg/kg) (E+L-NAME) killed 50 days after the first immunization. A) Seminiferous tubules (ST) with normal spermatogenesis in N rats. Testis section from a rat of E+saline group, shows all ST with different degrees of germ cell sloughing and moderate interstitial lymphomononuclear infiltrates (focal EAO). ST with moderate germ cell sloughing intermingled with ST with normal spermatogenesis were observed in rats from E+L-NAME group. Scale bar indicates 100μm. B) EAO score of testes from rats of N (n = 9), E+saline (n = 11) or E+L-NAME (n = 12), groups. Horizontal lines represent the mean, the dotted line indicates EAO score for values above 2.5. Each symbol represents a single rat. EAO score (mean±SEM): N: 0.111 ± 0.09, EAO+saline: 4.273 ± 0.821, E+L-NAME: 1.667, p<0.01. Student t Test. C) % of ST with apoptotic germ cells (GC, TUNEL technique). Each bar represents mean±SEM. **p<0.01 vs N and ^Δ^p<0.05 vs E+saline. Bonferroni Multiple Comparison Test. D) Nitrite content was evaluated in the testis of N, E+saline and E+L-NAME rats. Each bar represents mean±SEM. **p<0.01 vs N and ^Δ^p<0.05 vs E+saline. Bonferroni Multiple Comparison Test.

Histopathology of testes of E+saline treated rats presented numerous ST with germ cell sloughing and spermatogenesis impairment (Fig 2A). In the testis of L-NAME treated rats moderate germ cell sloughing was observed in some ST intermingled with other ST with normal spermatogenesis (Fig 2A). None of the N rats showed pathological alterations of the testis (Fig 2A). A 38.630±9.906% of damaged ST in rats from E+saline group compared to 8.516±1.334%, in rats from E+L-NAME group was detected (mean±SEM, n = 11–12, Student t Test, p<0.05). L-NAME treatment of E rats significantly reduced the EAO score as well as EAO incidence (64% in the E+saline group compared to 8% in the E+L-NAME group) (Fig 2B). We also observed that L-NAME protected germ cells from death since the percentage of ST with apoptotic germ cells was significantly reduced in the E+L-NAME group compared to E+saline (Fig 2C). Importantly the effects of L-NAME occurred concomitantly with a significant reduction in testicular nitrite content (Fig 2D). We also observed increased testosterone content in the testis of E+saline compared to N rats. Chronic treatment with L-NAME reduced the rise of intratesticular testosterone levels whereas no significant variations in LH and FSH serum levels were detected in all experimental conditions (Table 1).

**Table 1:** Serum testosterone (T), FSH and LH levels and testicular T content in untreated normal rats or immunized with TH and adjuvants (E) and injected with saline or L-NAME.

Group	Se rum T (ng/ml)	Intratesticular T (ng/gr testis weight)	Se rum LH (ng/ml)	Se rum FSH (ng/ml)
Normal	1.300±0.490	4.085±1.723	0.817±0.175	5.964±0.561
E+saline	1.068±0.199	11.717±2.340*	0.512±0.071	4.522±0.454
E+L-NAME	0.872±0.120	4.517±1.083^ΔΔ^	0.442±0.105	4.624±0.561

Hormone levels were measured by RIA in sera and testes from untreated normal rats (n = 9) or rats immunized with TH and adjuvants and injected with saline (E+saline, n = 11) or L-NAME (E+L-NAME, n = 12). Data are expressed as mean±SEM.

*p<0.01 versus respective normal and

^ΔΔ^ p<0.05 vs. E+saline.

Dunn Multiple Comparison Test.

### Nitric oxide released by testicular macrophages is involved in germ cell apoptosis and testosterone secretion

**Fig 3:**
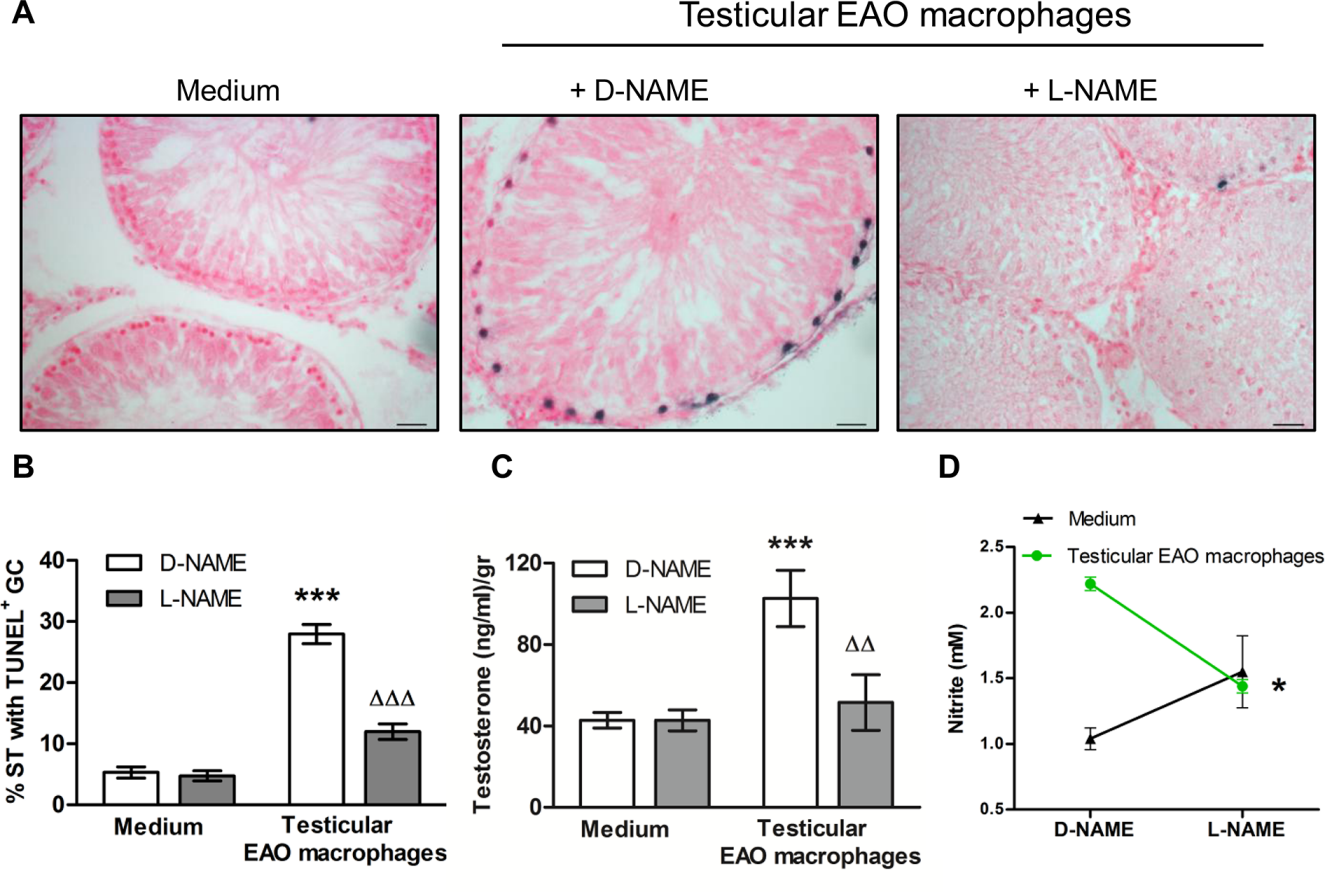
Effect of NO released by testicular macrophages on germ cell (GC) apoptosis (TUNEL technique) and testosterone secretion (RIA). A) Representative microphotographs of sections of testicular fragments (TF) from normal rats incubated with medium alone or testicular macrophages (TM) isolated from EAO rats in the presence of D-NAME (2mM) or L-NAME (2mM) for 18h; apoptotic GC are blue stained. Scale bar indicates 20μm B) % of seminiferous tubules (ST) with apoptotic GC, the % of ST with apoptotic cells in the TF immediately removed from the testis was 3.140±0.890. Data represent mean±SEM of (n = 30–40) non consecutive sections of TF obtained from four animals in four independent experiments. 300 ST were counted in each condition/rat. ***p<0.001 vs medium D-NAME; ^ΔΔΔ^p<0.001 vs D-NAME TM. Two-way ANOVA followed by Bonferroni Multiple Comparison Test. C) Testosterone content in the media. Data represent the mean±SEM (n = 5–7 wells/group from two experiments); ***p<0.001 vs medium D-NAME, ^ΔΔ^ p<0.01 vs D-NAME TM. Two-way ANOVA followed by Bonferroni Multiple Comparison Test. D) Nitrite production measured in the medium (fluorometric kit). Data represent mean±SEM (n = 5–7 wells/group from two experiments). *p<0.05 vs D-NAME TM. Student t Test.

To analyze whether NO released by EAO testicular macrophages was involved in germ cell death and testosterone release from Leydig cells, TF from N rats were co-cultured with or without testicular macrophages isolated from rats with orchitis. We found a significant increase in the percentage of ST bearing apoptotic germ cells which were localized at the basal compartment (Fig 3A and 3B). Increased production of nitrite generated by EAO testicular macrophages was significantly decreased in the presence of L-NAME (Fig 3D). To highlight the relevance of pro-inflammatory macrophages in the apoptotic effect on germ cells, TF were co-cultured with macrophages obtained from N (normal, non inmunized rats). Incubation of TF with macrophages obtained from N rats did not increase the percentage of ST with apoptotic germ cells compared to TF incubated with medium (TF+N macrophages 6.178±0.904, TF+medium: 5.310±0.910; mean±SEM of n = 30–40 non consecutive sections of TF obtained from four animals in four independent experiments, 300 ST were counted/rat.). Nitrite production by N testicular macrophages was significantly lower than that produced by macrophages from EAO rats (N macrophages: 1.270±0.057 μM vs. EAO macrophages: 2.220± 0.036, p<0.05 mean±SEM, n = 5).

Testosterone content in the culture medium significantly increased when TF were co-cultured with macrophages obtained from EAO rats compared to medium alone, and the presence of L-NAME reduced testosterone production by Leydig cells to levels similar to those found in TF incubated with medium alone (Fig 3C).

### NO induces germ cell apoptosis through activation of external and internal pathways

**Fig 4:**
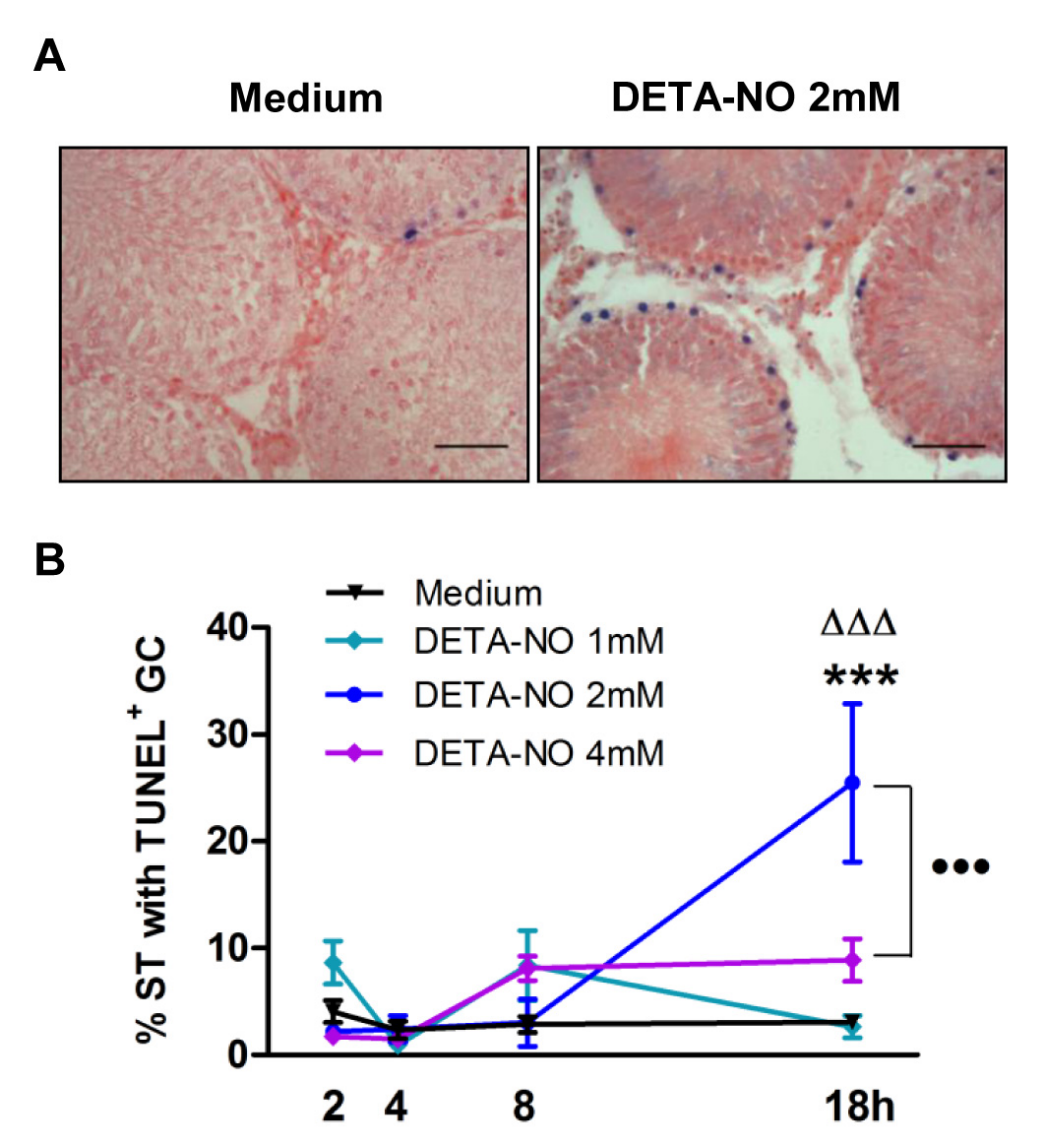
Evaluation of germ cell (GC) apoptosis in testicular fragments (TF) from normal rats incubated with DETA-NO (TUNEL technique). A) Upper panel: representative microphotographs of sections of TF obtained from normal rats incubated with medium containing or not DETA-NO (2mM) for 18h; apoptotic germ cells are blue stained. The scale bar indicates 50μm. B) Apoptosis evaluated on sections of TF. Each point represents mean±SEM of (n = 20–24) non consecutive sections of TF obtained from two rats in two independent experiments. 300 seminiferous tubules (ST) were counted in each experimental condition/rat.***p<0.001 vs. medium, ^ΔΔΔ^p<0.001 vs. DETA-NO 2mM at 2, 4 and 8h; •••p<0.001 vs. DETA-NO 4mM 18h. Bonferroni Multiple Comparison Test.

**Fig 5:**
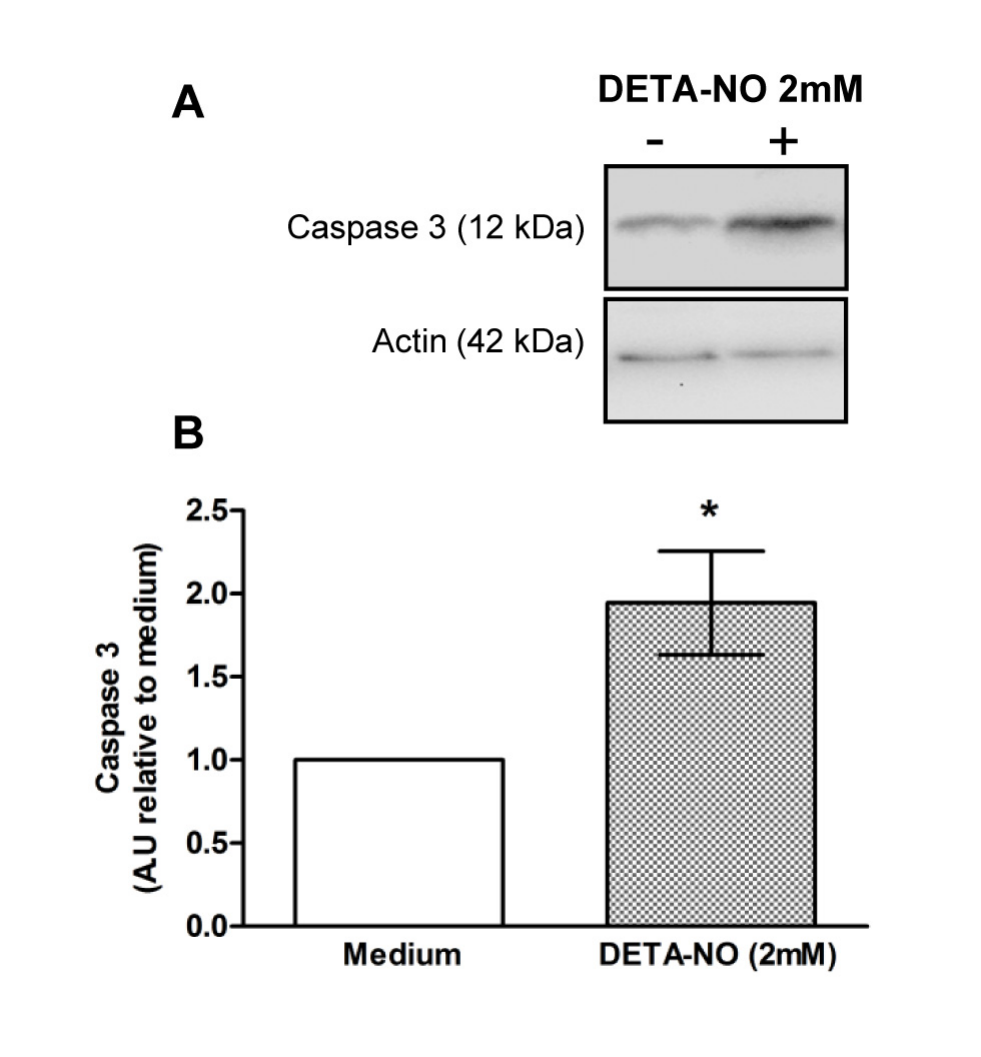
Effect of DETA-NO on caspase 3 activation. Testicular fragments obtained from normal rats were incubated with medium containing or not DETA-NO for 18h. A) Representative Western blot of caspase 3 content in cytosolic lysates B) Semiquantitative results of caspase 3 fragment content obtained by densitometric analysis of Western blots. A.U.: arbitrary units. Data from medium were arbitrarily set at 1. Each bar represents mean±SEM of 10 rats. *p<0.05 vs. medium. One sample t Test.

**Fig 6:**
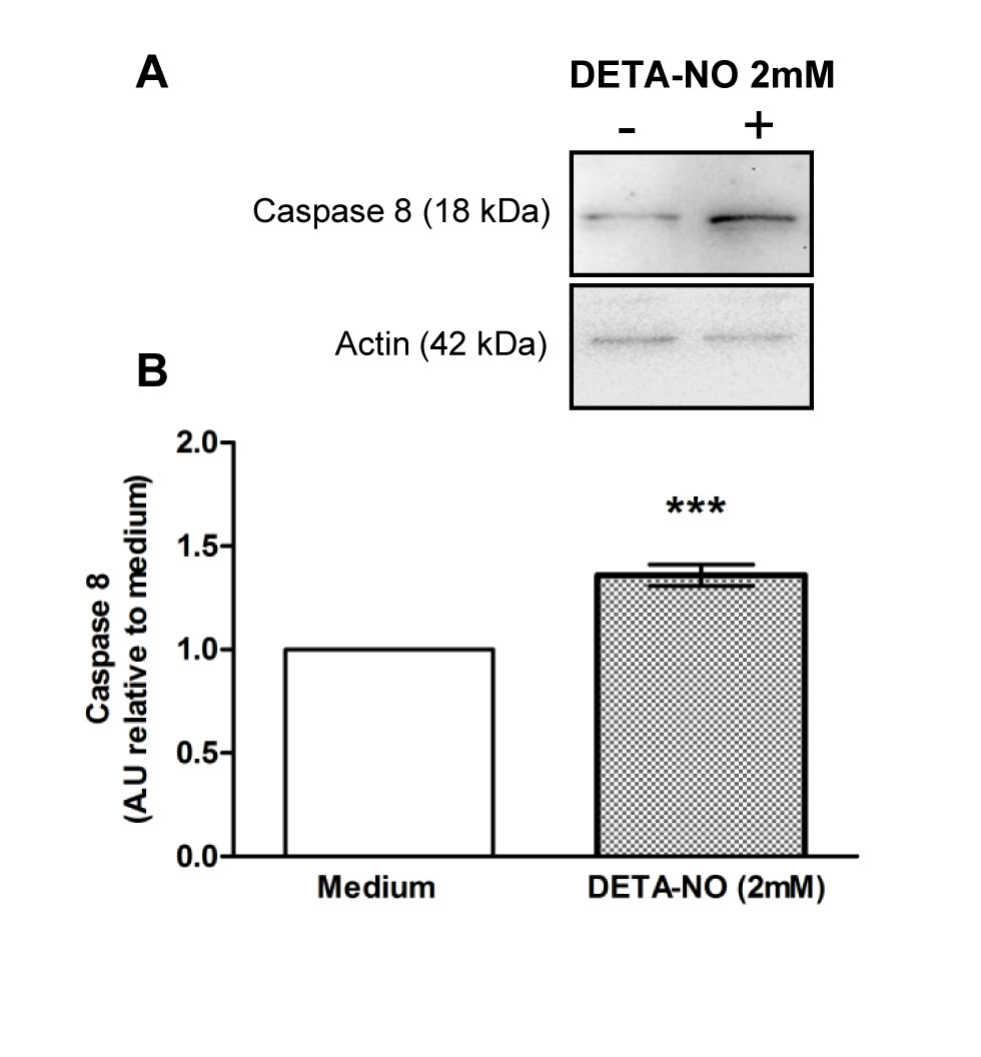
Effect of DETA-NO on caspase 8 activation. Testicular fragments obtained from normal rats were incubated with medium containing or not DETA-NO for 18h. A) Representative Western blot of caspase 8 content in cytosolic lysates B) Semiquantitative results of caspase 8 fragment obtained by densitometric analysis of Western blots. A.U.: arbitrary units. Data from medium was arbitrarily set at 1. Each bar represents mean±SEM of 10 rats. *** p<0.01 vs. medium. One sample t Test.

TF from N rats were incubated with different doses of DETA-NO for 2, 4, 8 and 18h. DETA-NO significantly increased the percentage of ST bearing apoptotic cells after 18h incubation with 2 and 4mM concentrations and was highest at 2mM (Fig 4B). Apoptotic germ cells were localized in the ST basal compartment. Also, Western blot of DETA-NO treated TF revealed a rise in caspase 3 (two fold) and caspase 8 activation compared to medium alone (Figs 5 and 6).

**Fig 7:**
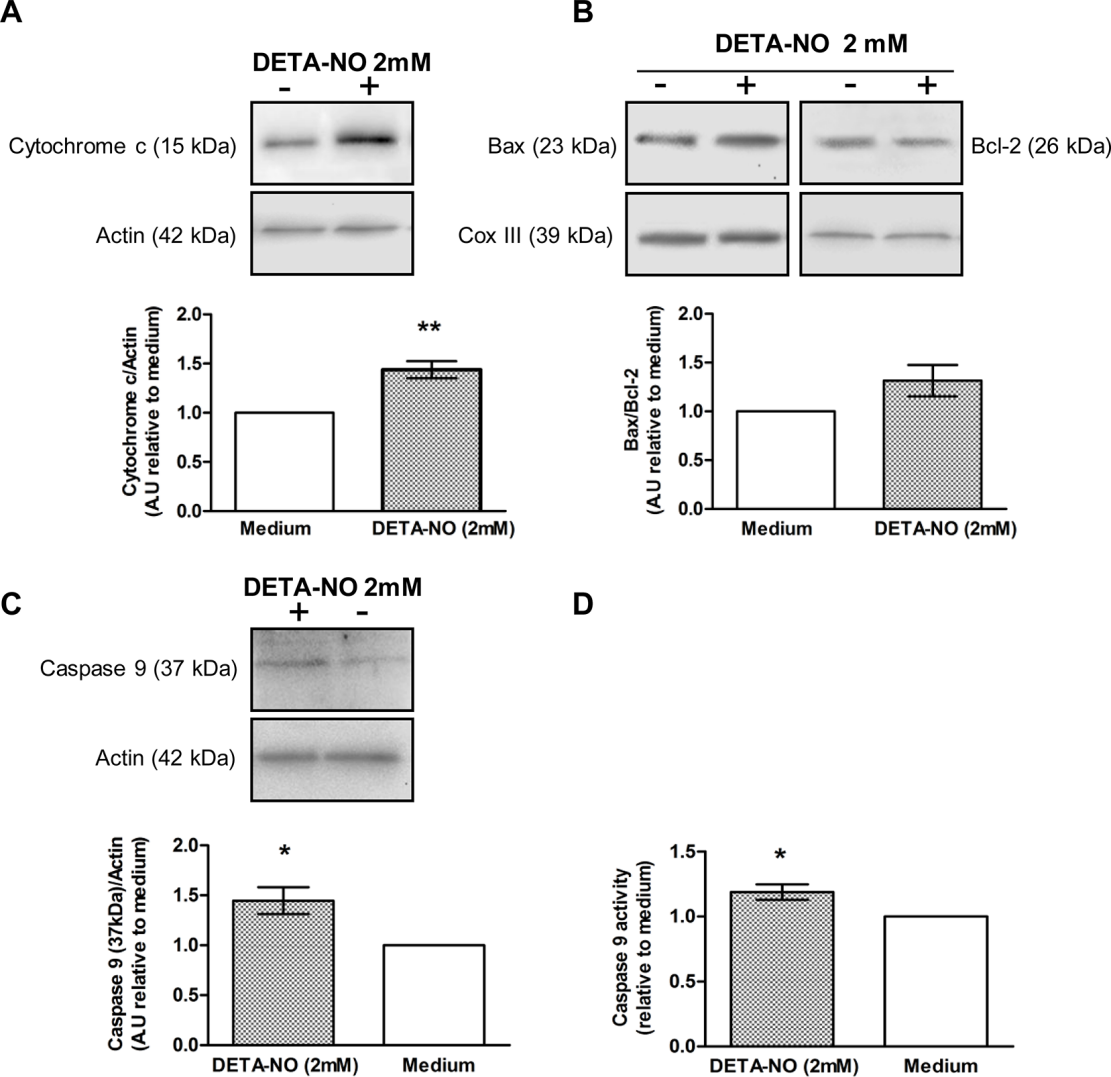
Effect of DETA-NO on apoptotic mitochondrial pathway activation. Testicular fragments obtained from normal rats were incubated with medium containing or not DETA-NO for 18h. Representative Western blots and semiquantitative results obtained by densitometric analysis of Western blots. A.U.: arbitrary units. Data from medium were arbitrarily set at 1. A) Cytochrome c content in the cytosolic fractionand B) Ratio of Bax/Bcl-2 content in the mitochondrial fraction (n = 10 rats) C) Caspase 9 processing and D) activity (colorimetric assay) in the cytosolic fraction (n = 10 rats). Each bar represents mean±SEM. *p<0.05 and **p<0.01 vs. medium. One sample t Test.

DETA-NO significantly increased cytochrome c content in the cytosolic compartment of the TF (Fig 7A). Since cytochrome c released from mitochondria to cytosol may be mediated by changes in the balance of Bcl-2 family proteins in the organelle, we analyzed Bax and Bcl-2 content (Fig 7B). Overall results showed that DETA-NO did not significantly affect the Bax/Bcl-2 ratio. Concording with the rise in cytochrome c, the content of the 37 kDa fragment of caspase 9, generated by caspase 3 protease activity [20], increased in the cytosolic fraction of TF incubated with DETA-NO (Fig 7C), concomitantly with caspase 9 activity (Fig 7D).

**Fig 8:**
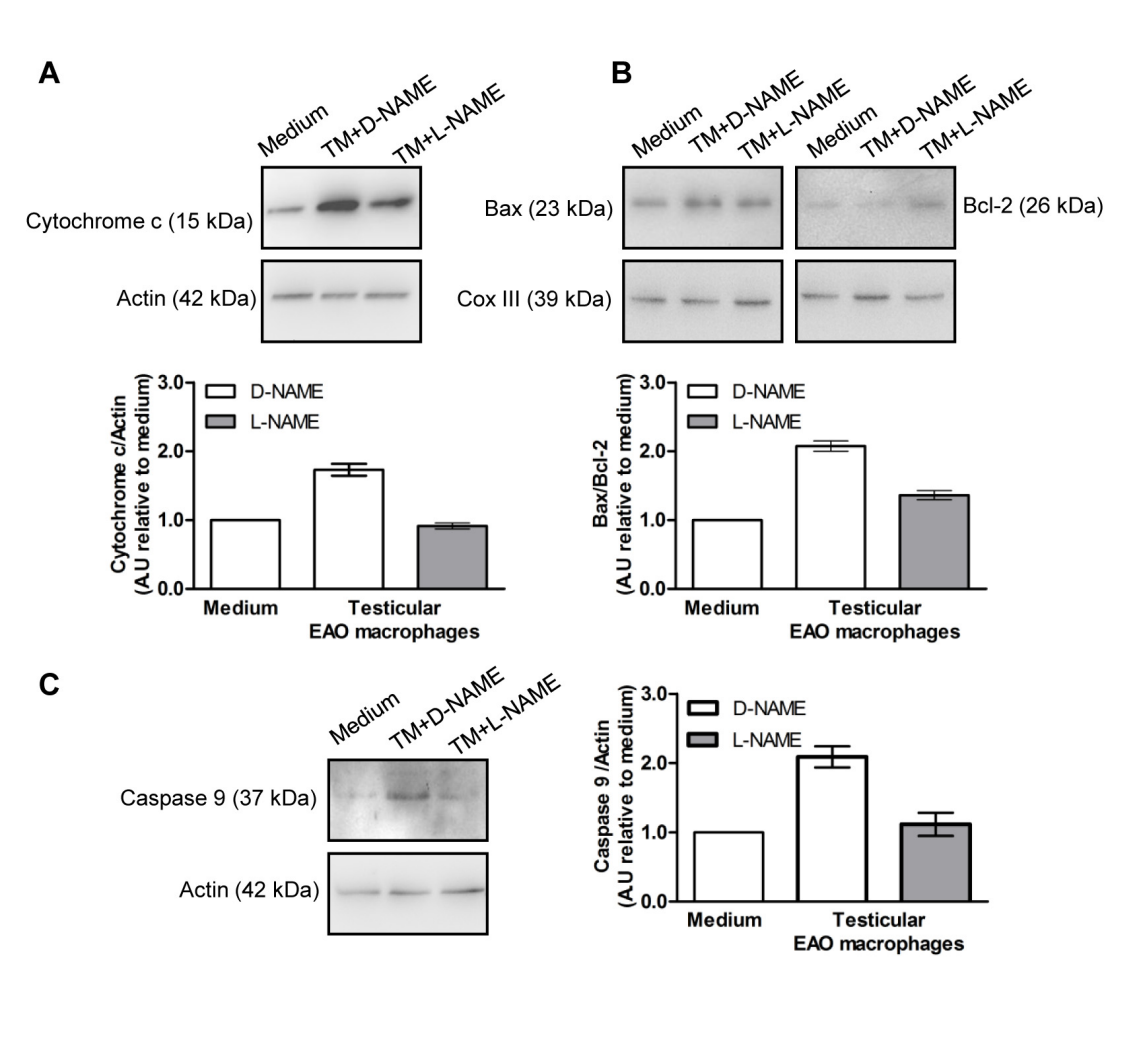
Effect of NO released by testicular macrophages on apoptotic mitochondrial pathway activation. Testicular fragments from normal rats incubated with medium alone or testicular macrophages (TM) isolated from EAO rats in the presence of D-NAME (2mM) or L-NAME (2mM) for 18h. Representative Western blots and semiquantitative results obtained by densitometric analysis of Western blots. A.U.: arbitrary units. Data from medium were arbitrarily set at 1. A) Cytochrome c content in the cytosolic fraction. B) Ratio of Bax/Bcl-2 content in the mitochondrial fraction. C) Caspase 9 processing in the cytosolic fraction. Each bar represents mean±SEM of three rats in three independent experiments.

We also evaluated the activation of the mitochondrial pathway in TF co-cultured with EAO macrophages in the presence or not of L-NAME (Fig 8). Similarly, in TF incubated with EAO macrophages we observed a two-fold increase in cytochrome c content and in the 37 kDa caspase 9 fragment in the cytosolic fraction compared to TF incubated with medium alone; moreover this effect was not observed when macrophages were incubated with L-NAME (2mM) (Fig 8A and 8B). Bax/Bcl-2 ratio was higher in the mitochondrial fraction of TF incubated with EAO macrophages compared to medium; this effect was reduced in the presence of L-NAME (Fig 8C).

### Oxidative stress is a mediator of NO effect on apoptosis

Over production of NO may cause oxidative stress and lipid peroxidation [21] We evaluated lipid peroxide generation in the testis of N and E rats Lipid peroxide content was elevated in the testis of E compared to N rats (mean±SEM N:6.079±0.371 μM/mg protein (n = 8), E:8.486±0.891 (n = 11) p<0.05 Student t test).

**Fig 9:**
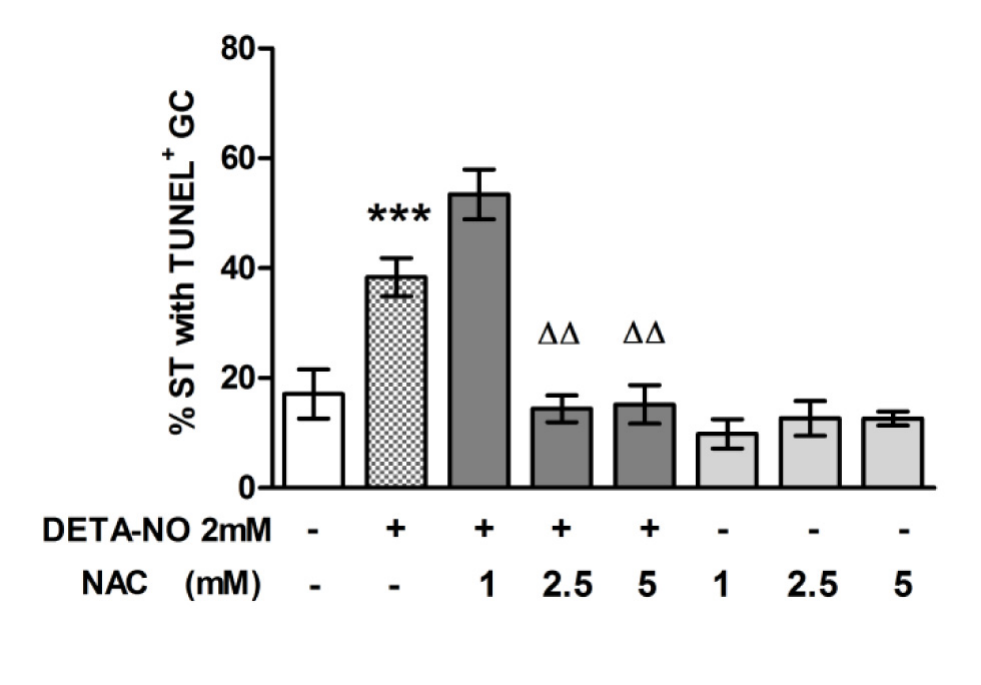
Involvement of oxidative stress in the apoptotic effect of DETA-NO on germ cell (GC) apoptosis. A) Testicular fragments (TF) obtained from a normal rat were incubated with medium containing or not DETA-NO in the presence or absence of NAC for 18h. Apoptosis was evaluated by TUNEL technique on sections of TF. The % of seminiferous tubules (ST) with apoptotic cells in the TF immediately removed from the testis was 2.482±0.967; data represent mean±SEM of n = 6–9 non consecutive sections of TF from one representative experiment of three. 300 seminiferous tubules were counted in each condition. ***p<0.001 vs. medium and ^ΔΔ^p<0.01 vs. DETA. Bonferroni Multiple Comparison Test.

To determine whether NO effect on germ cell apoptosis was mediated by reactive species that generate oxidative stress, we used a general antioxidant, N-acetyl-L-cysteine (NAC) that may act per se or as a precursor of glutathione synthesis. The presence of NAC (2.5 mM and 5 mM) in the culture medium prevented the increase in the percentage of ST with apoptotic germ cells induced by DETA-NO (Fig 9). The percentage of ST with apoptotic germ cells was similar in TF incubated with medium alone or with NAC at all concentrations studied.

### NO inhibited testosterone secretion from testicular fragments

**Fig 10:**
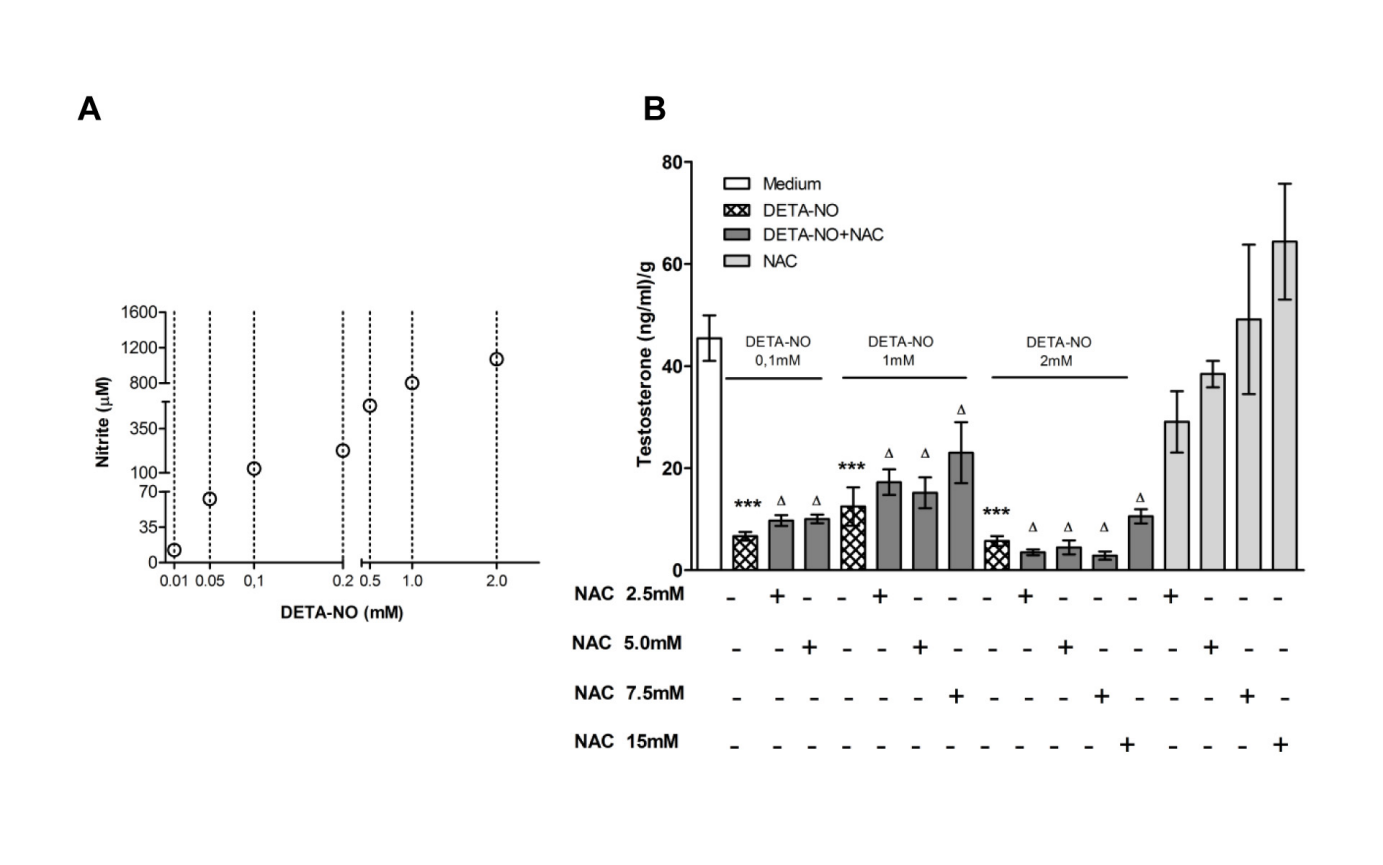
Effect of DETA-NO on testosterone secretion. A) Determination of nitrites generated from different concentrations of DETA-NO in DMEM/F12 medium without phenol red. Each circle represents mean of three wells (B) Testicular fragments obtained from normal rats were incubated with media containing or not DETA-NO in the presence or absence of NAC for 18h. Testosterone content in the media was evaluated by RIA. Each bar represents mean±SEM (n = 5–8 rats) from two independent experiments. ***p<0.001 and ^Δ^p<0.05 vs. medium. Bonferroni Multiple Comparison Test.

We evaluated the effect of DETA-NO from 0.1 mM to 2 mM which generates nitrite levels from 60 μM to 0.1mM when Leydig cell environment is preserved as occurs in the TF (Fig 10A). We observed that DETA-NO at all concentrations significantly inhibited basal testosterone secretion from Leydig cells present in the TF (Fig 10B). The presence of NAC in the culture medium did not prevent the inhibitory effect of DETA-NO (Fig 10B) although NAC significantly reduced nitrite levels in the incubation media (μM/gr TF, DETA-NO 0.1 mM: 253.296±228.670, DETA-NO 0.1 mM+NAC 5 mM: 134.668±17.957, p<0.05, mean±SEM, n = 5 rats).

## Discussion

Oxidative stress has been largely associated with male infertility. Many studies done in experimental models have shown up-regulation of the pro-oxidative enzyme NOS in pathological conditions of the testis. However, direct evidence of the contribution of NO-NOS system to testicular impairment in a chronic inflammatory disease is lacking. As we previously demonstrated, in rats with autoimmune orchitis up-regulation of NOS activity and expression occurs associated with high levels of testicular NO, spermatogenesis impairment and alteration of testosterone secretion [12,14]. This prompted us to evaluate the involvement of NO in the induction of pathological alterations that occurs in EAO.

Over production of NO during orchitis generates oxidative stress as we demonstrated by the increased testicular content of lipid peroxides, indicating an imbalance between the production of reactive nitrogen species and the antioxidant defense in the inflamed testis. To reduce testicular impairment associated with up regulation of pro-oxidative NOS we administrated L-NAME, a competitive inhibitor of NOS, to experimental rats undergoing orchitis. L-NAME decreased the incidence and severity of orchitis and reduced testicular NO production protecting germ cells from apoptosis. Concordantly, other authors previously demonstrated that a NOS inhibitor, N^G^-monomethyl-L-arginine, reduced sinovia inflammation in autoimmune arthritic rats and prevented glomerulo-nephritis development in multiple spontaneous autoimmune diseases in mice (MRL-lpr/lpr) [22,23]. In relation to the increased steroidogenic activity observed in the testis of EAO rats, also demonstrated by Suescun et al (1994) [12], chronic treatment with L-NAME prevented the rise of intratesticular testosterone content without affecting serum LH concentration, indicating local action of NO on testosterone secretion. We observed that in rats with orchitis high levels of NO occur concomitantly with increased intratesticular testosterone content. We also demonstrated that DETA-NO reduced testosterone secretion from Leydig cells present in TF cultures, which tallies with the reported inhibitory effect on testosterone release from Leydig cell cultures of different NO donors such as SNP, DEA-NO, SNAP and also DETA-NO [24–26]. DETA-NO concentration used in the in vitro experiments generated levels of nitrites in the range of those produced during orchitis; however it is difficult to predict NO levels around Leydig cells since they are in close proximity to macrophages, the main NO producers in the testis. We speculate that modulation of NO action in an inflammatory microenvironment may induce a steroidogenic stimulatory instead of the inhibitory effect observed when normal TF were incubated with DETA-NO only.

The high intratesticular testosterone level observed in the course of chronic inflammation [12,27] may be the outcome of multiple factors: i.e., a) the increased number of Leydig cells [12], b) converging on-off cytokines signaling cascades modulating steroidogenic enzyme activity and/or expression and c) eventually, a decrease in testosterone metabolic clearance due to alterations in blood flow or capillary permeability. In addition to the presence of cytokines in the inflamed testis known to up-regulate (IL-1, IL-6, and TNF-alpha) or inhibit (IFN-γ) testosterone secretion from Leydig cells [28–30] NO may also be involved in such regulatory mechanism since reduction of NO levels (by L-NAME treatment) restored testosterone levels to that of the normal testis.

Recently, we demonstrated that EAO interstitial macrophages express NOS enzymes and generate high levels of NO [14]. In the present study we ran co-cultures of TF from normal rats with testicular EAO macrophages to elucidate the pathogenic role of NO produced during testicular inflammation. We demonstrated that NO released by EAO testicular macrophages induces germ cell apoptosis, activating the mitochondrial pathway and contributing to support high testosterone secretion from Leydig cells. Although the presence of L-NAME in co-cultures reduces NO effect on germ cell apoptosis and restores testosterone production by Leydig cells, we cannot exclude the role of other factors released by testicular macrophages. In fact, molecules with a molecular weight of less than 20 kDa, able to pass through the 0.4 μM pore of the PET insert, such as IL-4 (14 kDa), IL-13 (17 kDa) IL-1-β (17.5 kDa) and IFN-γ (17 kDa) may contribute to modulate germ cell apoptosis and steroidogenesis [31–33].

Many data report the effect of conditioned media (CM) of non-testicular macrophages on Leydig cell steroidogenesis [25,34,35] but very few have analyzed the action of activated testicular macrophages. Lombard-Vignon et al. (1991) [36] demonstrated that steroid production is increased in Leydig cells treated with CM obtained from lipopolysacharide treated testicular macrophages and not with un-stimulated testicular or peritoneal macrophages. Previous results from our group, which concord with other authors [29], demonstrated a biphasic effect of CM of testicular macrophages from rats with orchitis. Proportions of CM under 35% stimulated testosterone secretion from cultured Leydig cells whereas in a proportion of 50% or more they inhibited hormone secretion [37].

To explore the direct effect of NO and mediators involved in germ cell apoptosis we run in vitro experiments demonstrating that NO induces germ cell apoptosis through activation of external and mitochondrial pathways. Imbalance of members of the Bcl-2 protein family on the outer mitochondrial membrane in favor of pro-apoptotic proteins (Bax, Bid, Bad and Bak) provokes cytochrome c release and mitochondrial pathway activation. In our experiments, Bax/anti-apoptotic Bcl-2 ratio did not change in the mitochondria in response to a direct action of NO. Changes in Bax/Bcl-2 content in the mitochondria might be an earlier event than cytochrome c release [38] although many reports also revealed that these two phenomena occur simultaneously in response to NO [39,40]. NO or its derivatives may alter mitochondrial membrane permeability, opening the mitochondrial transition pore complex by direct physical interaction [41]. NO may also target p53 to mitochondria causing cytochrome c release to the cytosol, upon activation by S-nitrosylation and/or phosphorylation [42]. However in the microenvironment generated by EAO macrophages NO provokes an increase in Bax/Bcl-2 ratio simultaneously with cytochrome c release, which highlights the relevance of other cytokines as modulators of NO action as mentioned above.

Results indicate that germ cells and interstitial cells display different sensitivities to NO levels generated in the testis. Whereas lower NO levels inhibited steroidogenic activity in Leydig cells, higher levels were necessary to overcome death protective mechanism in germ cells. Although NO impaired Leydig cell function, it was not apoptotic for these cells at any concentration analyzed. NO at physiological concentrations (1–100 nM) is relatively un-reactive [6]. However, at higher concentrations NO may be converted into a number of more reactive derivatives that may oxidize and nitrosate cellular proteins. To analyze whether NO effect was mediated by oxidative stress we used N-acetylcysteine (NAC), a small molecule with antioxidant properties due to its ability to reduce thiol groups and also for being a precursor in the formation of the antioxidant glutathione [43]. In the presence of NAC the in vitro pro-apoptotic effect of NO was prevented, concording with a report of Maheswari et al. (2011) [44] showing that pro-apoptotic effects of H_2_O_2_ on cultured germ cells are inhibited by NAC. Since NAC did not prevent the inhibitory effect of NO on steroidogenesis, it is possible that residual NO and/or NO derivatives remained in the media sustaining its inhibitory action or that the inhibitory effect was not mediated by oxidative stress. Testosterone inhibition could have been related to the regulation of steroidogenic enzyme activity (P450scc and P450c17) by binding of NO to the Fe heme or to the thiol group of cystein present at the active site of the enzymes as proposed by Del Punta et al., (1996) [24], Pomerantz and Pitelka (1998) [25] and Ducsay and Myers (2011) [45]. We speculate that the former mechanism might be prevalent since NAC did not reverse the inhibitory effect of NO. It is also possible that p38 MAPK, a novel signaling pathway for oxidative stress mediating inhibition of steroid synthesis in Leydig cells, is involved in NO testosterone inhibition, since this pathway is activated by NO [46,47].

During EAO development spermatocytes and spermatids are the main targets of the immunological attack. However, we observed that DETA-NO and NO released by EAO macrophages induced apoptosis of basal germ cells of normal rats (spermatogonia and/or pre-leptotene spermatocytes). We speculate that pro-and anti-apoptotic protein expression may diverge in germ cells in a normal or inflamed microenvironment, resulting in cells with different sensitivities to apoptotic stimuli. In EAO, germ cells localized at the ST basal compartment expressed high levels of the anti-apoptotic Bcl-2 protein compared to those from normal testis [18]. Also, germ cells from rats with EAO are more sensitive to the apoptotic effect of pro-inflammatory cytokines such as TNF-α, IL-6 and Fas L compared to N rats due to increased expression of death receptors [37,48,49].

Oxidative stress plays a relevant role in the etiology of pathologies associated with testicular dysfunction and male infertility. Nonspecific anti-oxidants such as NAC and vitamin E have generally been used to treat the testis undergoing experimental oxidative stress and to improve semen quality, fertilization rates and pregnancy; however, their efficacy is still being studied [50–53].

To our knowledge, this is the first investigation that evaluates the effects of nitric oxide synthase inhibitor (L-NAME) on testicular function in rats suffering a chronic inflammatory process of the testis In summary, our results demonstrated that NOS-NO system plays a relevant role in development of chronic testis inflammation and that NO released by testicular macrophages participates in the induction of germ cell apoptosis and in modulation of Leydig cell testosterone secretion. Specific inhibition of the pro-oxidant up-regulated enzyme NOS in rats with orchitis by using L-NAME contributed to successfully improve testicular function. Our findings contribute to the understanding of mechanisms underlying testicular damage caused by oxidative stress and may be useful to develop new therapies for subfertility or infertility associated with chronic inflammatory processes by means of the use of nitric oxide synthase inhibitor.
